# Symbiotic relationship between brain structure and dynamics

**DOI:** 10.1186/1471-2202-10-55

**Published:** 2009-06-02

**Authors:** Mikail Rubinov, Olaf Sporns, Cees van Leeuwen, Michael Breakspear

**Affiliations:** 1 Black Dog Institute and School of Psychiatry, University of New South Wales, Sydney, Australia; 2 Mental Health Research Division, Queensland Institute of Medical Research, Brisbane, Australia; 3 CSIRO Information and Communication Technologies Centre, Sydney, Australia; 4 Department of Psychological and Brain Sciences, Indiana University, Bloomington, USA; 5 Laboratory for Perceptual Dynamics, RIKEN Brain Science Institute, Saitama, Japan; 6 Royal Brisbane and Women's Hospital Mental Health Service, Brisbane, Australia

## Abstract

**Background:**

Brain structure and dynamics are interdependent through processes such as activity-dependent neuroplasticity. In this study, we aim to theoretically examine this interdependence in a model of spontaneous cortical activity. To this end, we simulate spontaneous brain dynamics on structural connectivity networks, using coupled nonlinear maps. On slow time scales structural connectivity is gradually adjusted towards the resulting functional patterns via an unsupervised, activity-dependent rewiring rule. The present model has been previously shown to generate cortical-like, modular small-world structural topology from initially random connectivity. We provide further biophysical justification for this model and quantitatively characterize the relationship between structure, function and dynamics that accompanies the ensuing self-organization.

**Results:**

We show that coupled chaotic dynamics generate ordered and modular functional patterns, even on a random underlying structural connectivity. Consequently, structural connectivity becomes more modular as it rewires towards these functional patterns. Functional networks reflect the underlying structural networks on slow time scales, but significantly less so on faster time scales. In spite of ordered functional topology, structural networks remain robustly interconnected – and therefore small-world – due to the presence of central, inter-modular hub nodes. The noisy dynamics of these hubs enable them to persist despite ongoing rewiring and despite their comparative absence in functional networks.

**Conclusion:**

Our results outline a theoretical mechanism by which brain dynamics may facilitate neuroanatomical self-organization. We find time scale dependent differences between structural and functional networks. These differences are likely to arise from the distinct dynamics of central structural nodes.

## Background

Modular small-world network topology may represent a basic organizational principle of neuroanatomical connectivity across multiple spatial scales [1-6]. Small-world networks are clustered (like ordered networks), and efficiently interconnected (like random networks) [1]. Modular networks are characterized by the presence of highly interconnected groups of nodes (modules) [7]. Hence a modular small-world connectivity reconciles the opposing demands of segregation and integration of functionally specialized brain areas [8] in the face of spatial wiring constraints [9]. However the mechanisms underlying the emergence of small-world connectivity in a developing nervous system remain unknown. In this study, we utilize nonlinear dynamical and network analyses to shed light on such mechanisms. We do this by using a model which examines the influence of neuronal dynamics on the underlying structural connectivity.

Cortical structure and dynamics are highly interdependent. On relatively fast time scales, structure enables the emergence of complex dynamics [10]. On slower time scales dynamics act to reshape the structure via mechanisms such as activity-dependent dendritic development, synaptogenesis and synaptic pruning, as recently reviewed [11-13]. The influence of structural connectivity on neuronal activity is illustrated by the observation that profound disturbances in complex cognitive functions often result from relatively subtle disruptions in the underlying neuroanatomy, as for example in schizophrenia [14]. On the other hand, disruption of spontaneous activity in the developing cortex interferes, for instance, with specific axonal branching of pyramidal neurons [15]. Furthermore, detrimental effects of early visual deprivation illustrate the importance of spontaneous and sensory driven neuronal activity on circuit formation in the primary visual cortex [16].

There hence exists a "symbiotic" relationship between structural brain connectivity and brain activity. Such a relationship is thought to be central to the emergence of complex neuroanatomical connectivity from a relatively unstructured neuropil [17,18], and is increasingly examined computationally [19]. Previously, in a mathematical model of this relationship, random structural connectivity guided by emergent synchrony patterns was shown to evolve to modular small-world connectivity [20-22]. Here, we first advance the biophysical justification of this model, and then provide a detailed quantitative analysis of the relationship between structure, function and dynamics that accompanies the ensuing neuroanatomical self-organization.

The relationship between structural and functional brain connectivity is gaining rapid interest. Recent studies have explored this relationship by simulating neuronal dynamics on large scale neuroanatomical connectivity networks. These studies found that the resulting functional patterns passively reflect the underlying structural connectivity on slow time scales [23-25], but are significantly less constrained on faster time scales [25]. However, because the underlying structural connections were chosen *a priori *(from anatomical data) and were subsequently treated as static, these studies did not address the influence of activity upon structure, as mediated through dynamically driven structural plasticity. Such an influence forms the core of our investigation.

Several models of complex network growth have been well established in the wider network community. These include the well known preferential attachment model [26], as well as spatial growth models [27,28]. However, there has been much less focus on dynamically influenced network growth and plasticity [29]. In the brain network literature, dynamically driven network plasticity was implicitly implemented by Sporns et al. [30], who showed that a supervised search for structural networks exhibiting high functional complexity, retrieves cortical-like modular small-world connectivity. However, the algorithm of Sporns et al. is based on a supervised search for a "functionally optimal" topology from thousands of generated networks, and is consequently implausible in a maturing nervous system. Hence the mechanisms underlying the emergence of small-world cortical connectivity, and particularly the reciprocal influence of activity upon structure have been relatively unexplored.

**Figure 1:**
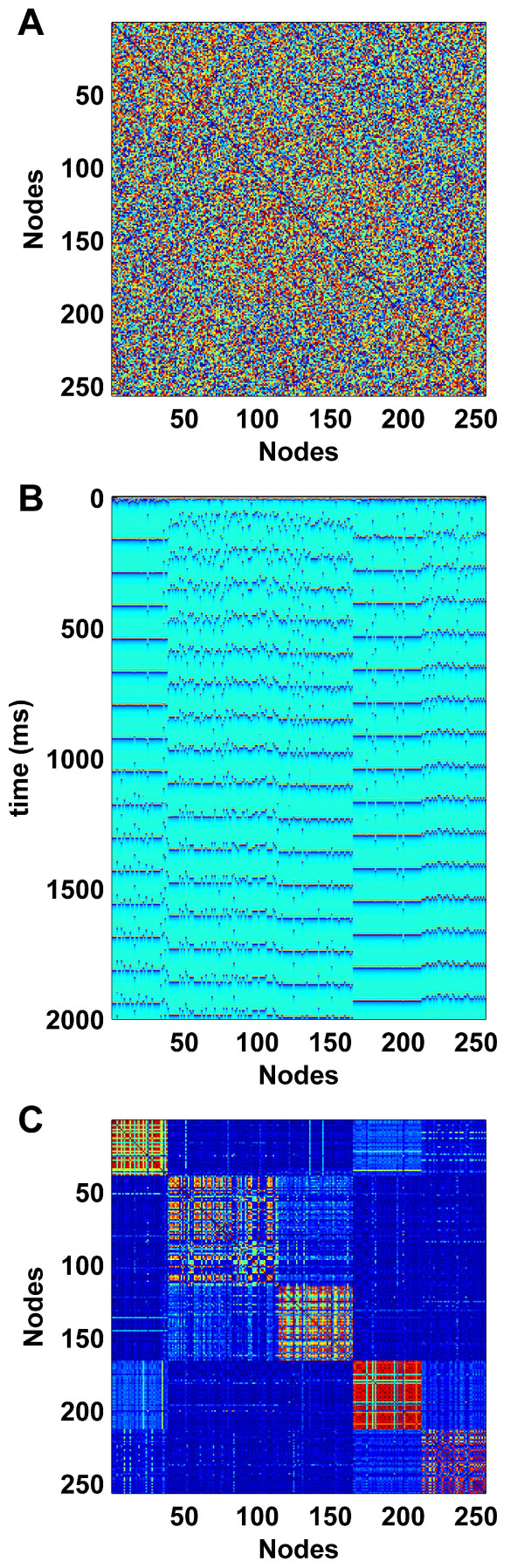
**Functional connectivity of simulated neural mass model dynamics on a random structural network**. (A). The underlying 256 node random structural network. Here and in the following figures, networks are represented by their square connectivity matrices, where row and column indices correspond to nodes, and matrix entries correspond to connections between individual nodes. (B). The emergent spatiotemporal dynamics: color represents the state of individual dynamical units according to space (horizontal axis) and time (vertical axis). (C). The resulting functional connectivity matrix, derived by linear cross-correlation between the spatiotemporal dynamics from B and reordered to maximize the visual appearance of modules (this reordering was also applied to A, with negligible impact). Each dynamical unit represents the mean state of a local population of densely connected inhibitory and pyramidal neurons, with conductance-based transmembrane ion flows and zero-order synaptic kinetics. Full details of these dynamics are provided in Breakspear et al. [33].

The nonlinear nature of neuronal dynamics [31] provides a foundation for an alternative, activity-dependent model. For instance, a conductance-based "neural mass" (i.e. population) model, developed to understand basic mechanisms of corticocortical coupling [32,33] was recently employed to simulate neuronal dynamics on a large scale structural connectivity matrix of the macaque [25]. This approach provided a novel explanation for the existence of two anticorrelated networks, as previously reported in human functional neuroimaging studies [34]. In contrast, Figure 1 shows the functional patterns that are generated by this same neural mass model on a random structural network, given as an image map in the top panel. The absence of modularity in a random network renders it unlike the known connectivity of the cortex. However, the spatiotemporal activity that unfolds on this structure (middle panel) evidences partial synchronization amongst the weakly and randomly coupled nodes, resulting in a modular functional connectivity matrix (bottom panel). This appearance of synchronous clusters in coupled nonlinear systems is a common feature of high-dimensional nonlinear systems [35]. It is intuitive to propose that the presence of functional modules will gradually, through activity-dependent synchrony-favoring rewiring, enable the emergence of similar modules in the underlying structural connectivity.

This intuition underlies the activity-dependent model of structural rewiring proposed by Gong and van Leeuwen [20] and further explored in this paper. This model simulates spontaneous cortical dynamics using coupled chaotic logistic maps, and gradually rewires the underlying structural connectivity towards the resulting synchrony patterns. The model hence represents a crude approximation of Hebbian learning in a spontaneously active – or "resting state" – ensemble of coupled oscillators. The learning is Hebbian as the connections are established between synchronous neurons and pruned between asynchronous neurons. The model hence simulates activity-dependent synaptic rewiring – an important mechanism of structural plasticity in the developing, as well as in the mature brain [13]. Note that synaptic rewiring is conceptually different to mechanisms of functional plasticity (potentiation or depression of synaptic weights), such as spike-timing dependent synaptic plasticity [36].

**Figure 2:**
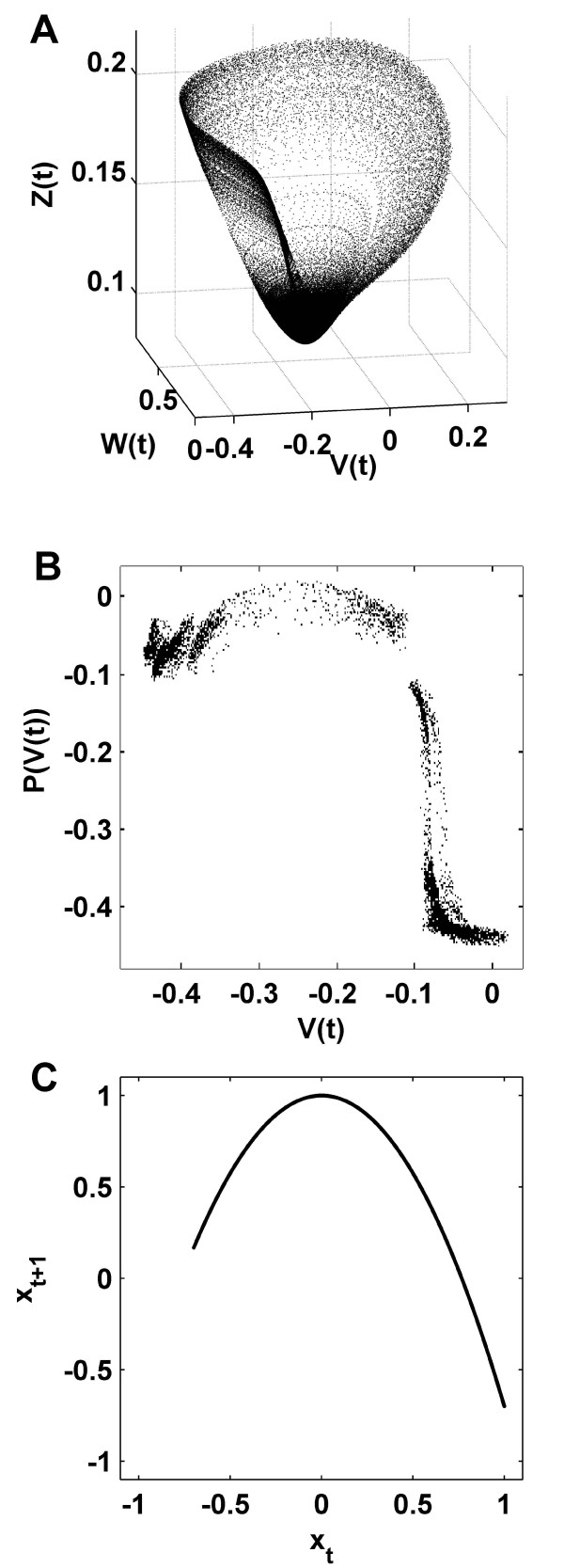
**Dimension reduction of nonlinear neuronal dynamics**. (A). Phase space attractor of a three-dimensional neural mass flow. This attractor is an illustration of the dynamics generated by the flow of a neural mass model (see Breakspear et al. [33]). The dynamical variables represent the mean membrane potential of pyramidal (*V*) and inhibitory (*Z*) neurons, and the average number of open potassium ion channels (*W*). (B). Poincaré first return map from the same attractor [33]; this map captures key features of the neural mass flow, by following each trajectory from one intersection (*V*) of the attractor to the next (*P*(*V*)). (C). The quadratic logistic map. This map has the same unimodal topology as the neural mass Poincaré return map. While the logistic map lacks the "thickness" of the neural mass map, it is several orders of magnitude faster to compute, hence allowing the detailed quantitative analysis in the present paper.

Consistent with the approach of Gong and van Leeuwen, the present study approximates neuronal dynamics using an ensemble of coupled chaotic unimodal maps. Such maps are well known to exhibit universal dynamical properties [37,38]. Hence generic properties of interacting nonlinear systems are well captured by networks of such simple maps. Importantly in a neuroscientific context, chaotic unimodal maps were recently used to model neuronal bursting behavior [39,40]. We previously reported [33] that unimodal maps are topologically similar to a Poincaré first return map of a chaotic neural mass model attractor (Figure 2). The first return map (Figure 2B) is a useful approximation of the full dynamics of the chaotic neural mass model. A major advantage of unimodal maps is their computational simplicity, which permits a detailed quantitative analysis of the mechanisms of self-organization, within a framework of general biophysical plausibility.

We hence seek a detailed exploration of the nature of this structural self-organization. We observe that, as in Figure 1, coupled chaotic dynamics generate ordered, modular functional patterns, even on random structural networks. Through the adaptive rewiring rule, structural topologies are reshaped by these patterns towards a modular small-world connectivity. We find that central hub nodes play a key role in the cohesiveness of this small-world network – the noisy dynamics of these hubs enable them to persist in structural networks despite ongoing rewiring and despite their comparative absence in functional networks.

## Results

### Interdependent evolution of structural and functional networks

Our model consists of an ensemble of chaotic logistic maps, coupled via a directed binary structural connectivity network. The dynamics of these maps generated a series of functional connectivity networks on static structural networks. As the dynamics evolved, structural networks were gradually adjusted towards emergent synchrony patterns: periodically, a node was randomly chosen and its connections were rewired such that it gained a link to a node with which it was most synchronous, and lost a link to a neighbor with which it was least synchronous. We measured synchronization using the absolute difference (Euclidean distance) between individual unit states (see Methods). We began simulations from initially random structural connectivity and proceeded until asymptotic conditions, as characterized by globally invariant structural and functional clustering and closeness.

**Figure 3:**
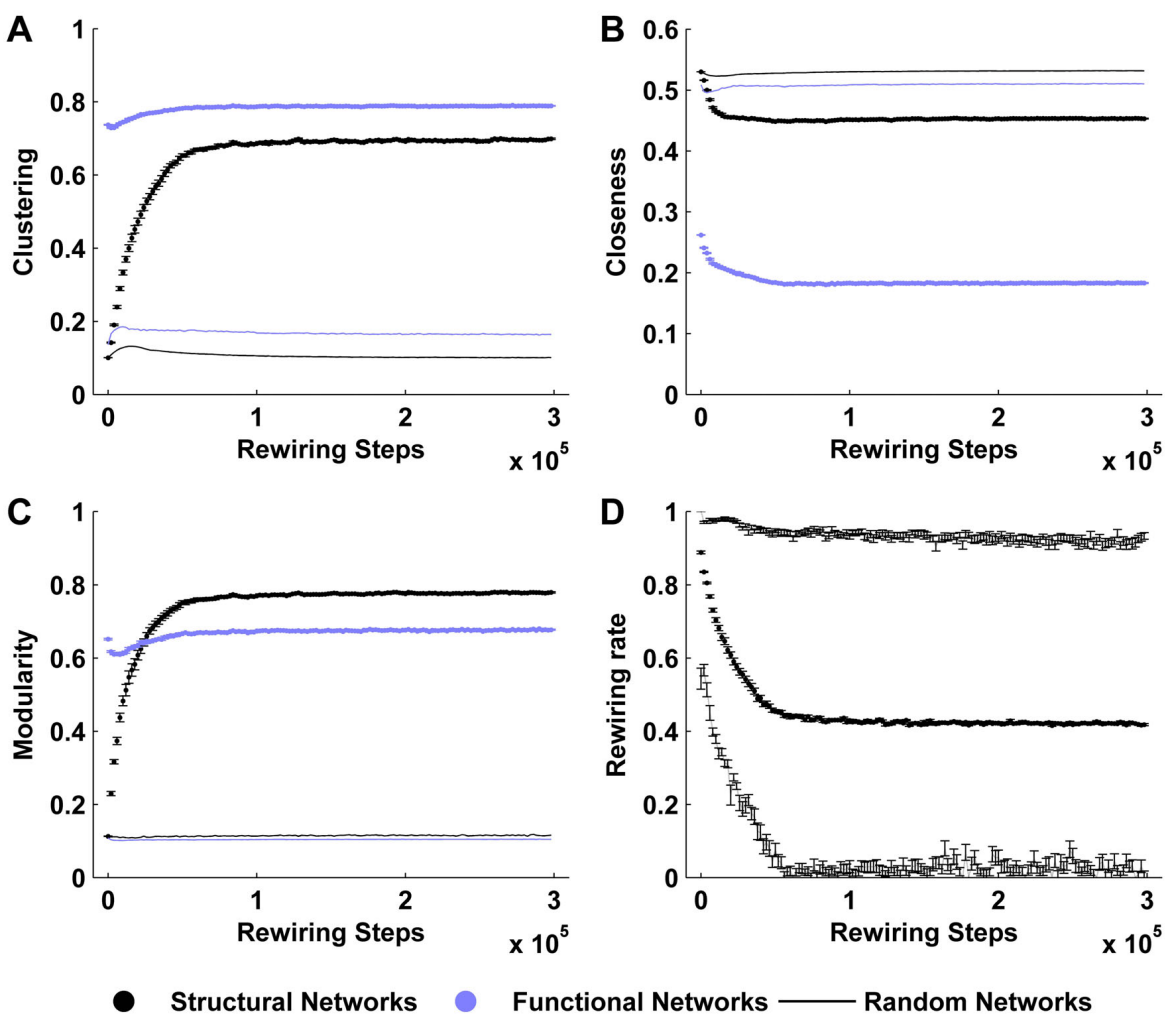
**Interdependent evolution of structural and functional networks**. Concurrent evolution of clustering (A), closeness (B) and modularity (C) of structural (black) and functional (blue) networks. Metrics derived from surrogate random networks (solid lines) are plotted for comparison. (D) Median, minimum and maximum rewiring rates at each rewiring step. While some nodes cease rewiring at the asymptotic state, others remain highly rewirable – hence rewiring is ongoing despite a stable structural topology. Error bars represent the standard error of the mean, as estimated over 20 simulations.

Figure 3 illustrates the interdependent evolution of structural and functional networks. Initial random structural networks are poorly clustered, weakly modular and highly interconnected. In contrast, the corresponding emergent functional networks are highly clustered, strongly modular and poorly interconnected. As the structural networks are iteratively reshaped towards functional networks, they acquire more ordered characteristics, as manifested by a steep rise in the clustering and modularity. Crucially, however, structural interconnectedness (closeness) remains comparable to that of surrogate random networks, enabling the emergence of a robust modular small-world topology. These changes in structural topology correspondingly reshape functional connectivity, as manifested by a small increase in functional clustering and reduction in functional closeness. Following a transient period of interdependent evolution, both structural and functional networks reach an asymptotic state despite ongoing structural rewiring.

**Figure 4:**
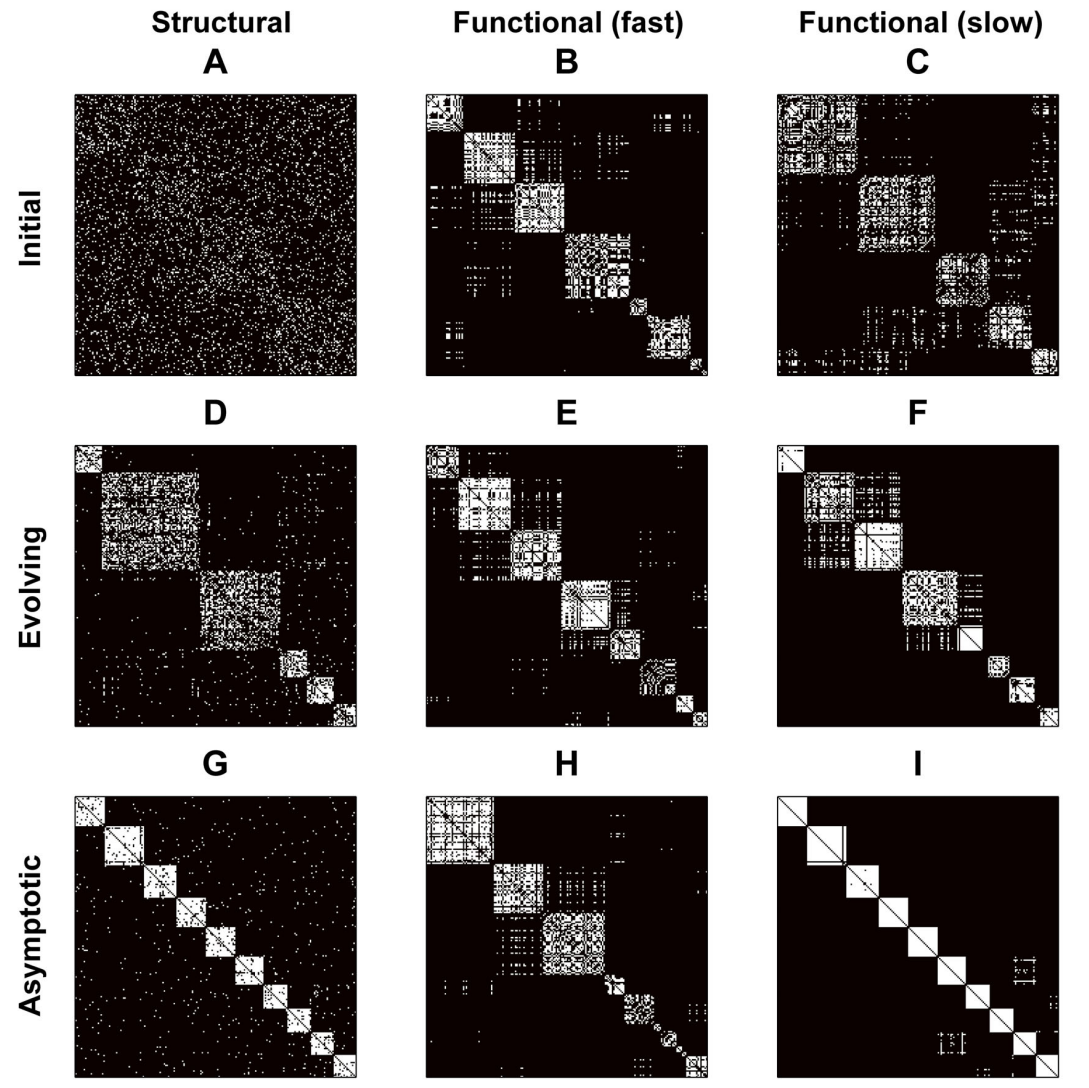
**Characteristic structural and functional networks at different phases in the evolution**. The initial (row 1), evolving (row 2) and asymptotic (row 3) network configurations are illustrated for structural (column 1), fast time scale (column 2) and slow time scale functional (column 3) networks. Fast time scale networks represent the instantaneous patterns of dynamical synchrony, measured as the Euclidean distance between individual unit states. Slow time scale networks are derived by calculating the correlation coefficient of 100 consecutive functional states. Nodes in all networks are reordered to maximize the appearance of modules, via the maximization of modularity (see Methods). Consequently, a network may be reordered differently, at different times in its evolution. However, given the similarity between structural and slow time scale functional networks, pairs (D)-(F) and (G)-(I) have exactly the same ordering in the current figure.

Figure 4 shows characteristic structural and functional networks at the initial, evolving and asymptotic states. For a given structural network (Column 1), we illustrate a typical fast time scale (Column 2), and slow time scale (Column 3) functional network. Nodes in all networks in this figure have been reordered to maximize the appearance of modules. Note that, as in Figure 1, random structural connectivity generates ordered functional connectivity. Evolving structural networks are characterized by the emergence of distinct modules, which reinforce a modular functional topology. However due to the continuous presence of weak inter-modular synchrony in asymptotic functional connectivity (Figure 4H), fast time scale functional networks are more clustered but less modular than their corresponding structural networks (Figure 3A,C).

A key difference between structural and functional connectivity is the robust presence of inter-modular links in structural networks, and a relative absence of these links in functional networks. Inter-modular links represent the crucial difference between a structural small-world and a functional lattice [1]. Below, we investigate the mechanisms underlying the persistence of these links in structural networks, by considering the distinct dynamics of central hub nodes.

The degree distributions in both structural and functional networks do not evolve toward a scale-free, or broad-scale distribution (Additional file 1). The presence of a scale-free degree distribution in structural brain connectivity remains controversial, chiefly because spatial constraints and high wiring cost are thought to impede such an organization [2,3,41].

### Robustness of structural self-organization

**Figure 5:**
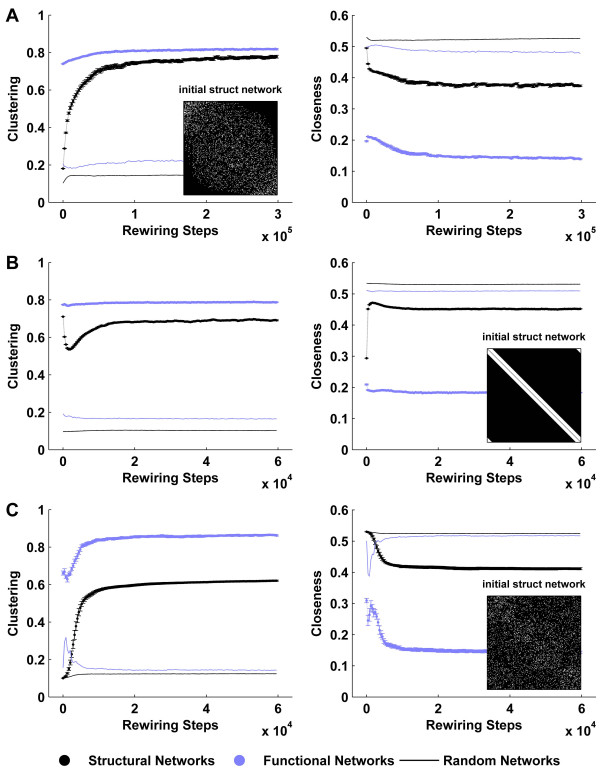
**Robustness of structural self-organization**. Temporal evolution of clustering and closeness of structural (black) and functional (blue) networks. (A) Evolution under spatial constraints (nodes are placed randomly on a three-dimensional sphere). (B) Evolution from an initial lattice structural topology. (C) Evolution under memory guided rewiring. Insets show initial structural connectivity matrices. Compared to Figure 3, the onset of a small-world topology is faster in (B) and (C) (note the difference in time scale). Metrics derived from surrogate random networks (solid lines) are plotted for comparison. Error bars represent the standard error of the mean, as estimated over 20 simulations.

We incorporated spatial constraints into our model by placing nodes randomly on the surface of a sphere, and subsequently restricting potential synapses to the spatially closest 40% of all neighbor pairs. Such an arrangement introduces some local clustering into the initial network topology (Figure 5A). Activity-dependent rewiring further increases such clustering, and importantly preserves high closeness, despite the spatial limitations. Hence, the resultant evolution is qualitatively equivalent to a non-spatially constrained topology. A similar result is obtained when the initial network was an ordered lattice, and no spatial constraints were imposed (Figure 5B). Given the initial lack of hubs in a lattice, it hence appears that hubs emerge during network evolution.

We evaluated the effects of incorporating a memory function into the rewiring rule, therefore effectively rewiring the system towards slow time scale functional networks (Figure 4, Column 3). An analysis incorporating a linear memory function (averaging 100 consecutive functional networks) likewise shows an evolution to a small-world structural network (Figure 5C). An equivalent evolution was also observed when networks were rewired at a fast learning rate (data not shown) – that is, when a rewiring was made at every iteration of the dynamics, instead of at every 1000 iterations.

**Figure 6:**
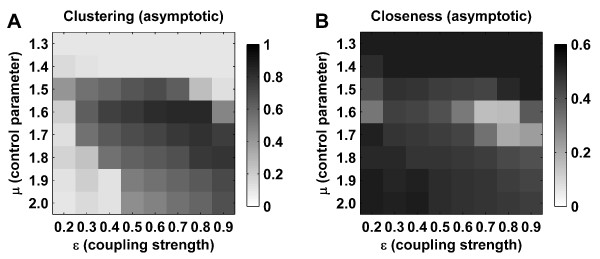
**Dependence of structural evolution on parameters**. Asymptotic values for structural clustering (A) and closeness (B), observed for a range of values of the control parameter *μ*, and coupling strength *ε*. Each matrix entry corresponds to the average asymptotic structural clustering or closeness for the corresponding parameter values. For example, asymptotic structural values from Figure 3A (≈ 0.7) and Figure 3B (≈ 0.45) are displayed under entries (*μ *= 1.7, *ε *= 0.5). Values of the control parameter at 1.4 and below correspond to periodic dynamics, and at 1.5 and above to chaotic dynamics. Evolution to a small-world network occurs under chaotic dynamics with moderate coupling. Values of clustering and closeness represent averages over 25 simulations of 500000 rewiring steps each.

We evaluated the dependence of the model on parameters by systematically varying the coupling parameter *ε *and the control parameter *μ *(see Methods). We hence evaluated structural evolution under a range of coupling strengths, and under periodic through to strongly chaotic dynamics. Figure 6 illustrates asymptotic structural clustering and closeness for different parameter values. Note that evolution to a small-world topology occurs across a large region of parameter space, as consistent with a previous exploratory analysis [20]. However the networks remain random-like under periodic dynamics (*μ *≤ 1.4), under weakly chaotic dynamics with strong coupling (e.g. *μ *= 1.5, *ε *= 0.9), or at the other extreme, under strongly chaotic dynamics with weak coupling (e.g. *μ *= 2.0, *ε *= 0.2). There also exists a small region of parameter space (e. g. *μ *= 1.6, *ε *= 0.8), under which the networks acquire ordered (highly clustered, but not close) topologies.

### Correlation between structural and functional network metrics

**Figure 7:**
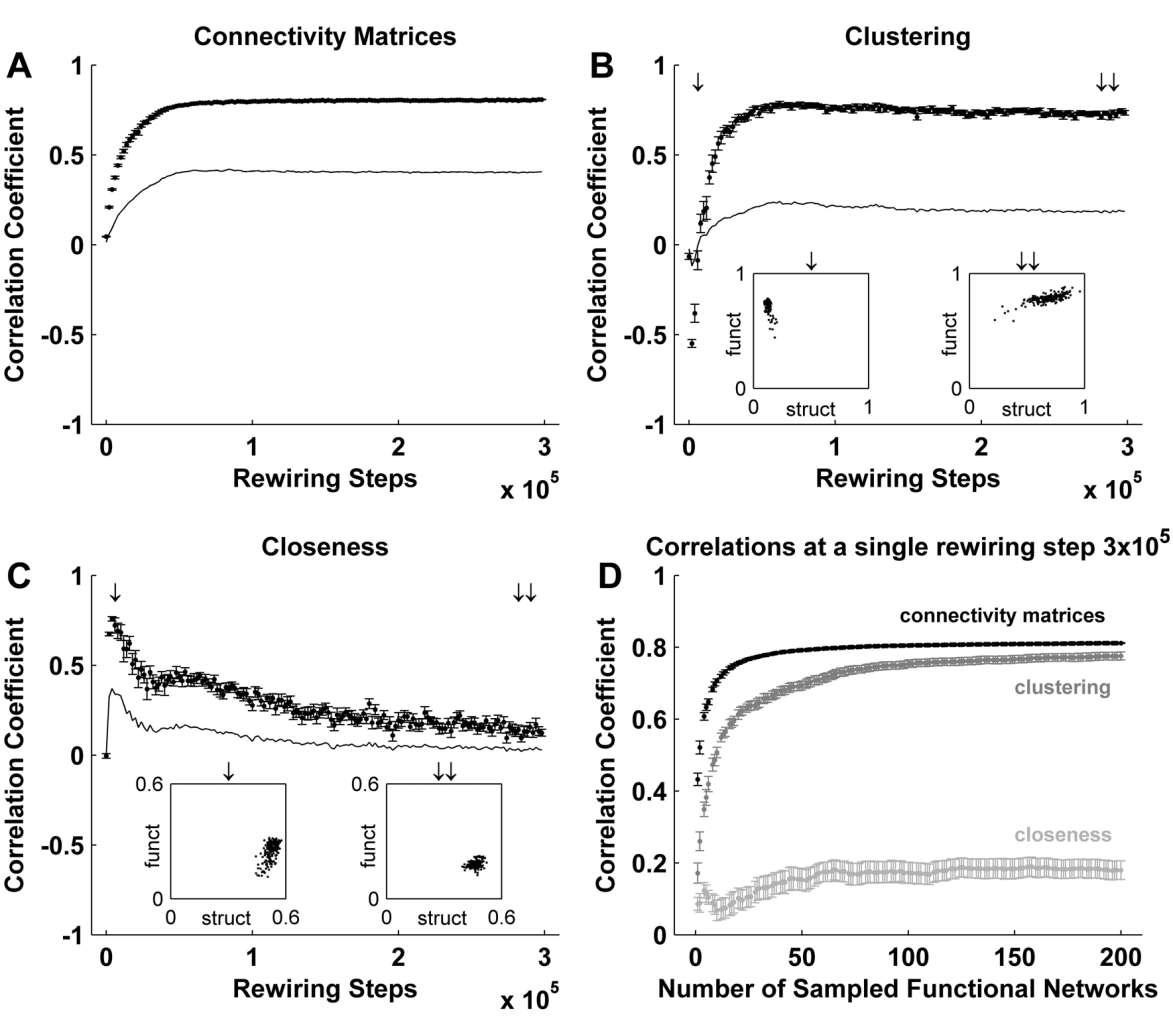
**Correlation between structural and functional network metrics**. Temporal evolution of the correlation coefficient between structural and functional networks (A), as well between node-wise structural and functional clustering (B) and closeness (C), with illustrative scatter plots (insets) at specified time instants. Functional network metrics are derived by analyzing fast time scale functional networks and averaging the resulting metrics (see main text for details). An alternative approach, which averages the correlations of fast time scale networks, results in significantly weaker correlations (solid lines). (C) Correlation between structural and functional clustering at a single structural state, plotted against the number of sampled instantaneous functional networks. A strong correlation emerges as more networks are sampled. Error bars represent the standard error of the mean, as estimated over 20 simulations.

We initially examined correlations between structural networks and averaged fast time scale functional networks. Figure 7A shows that these correlations are very high at the asymptotic state. In addition we examined node-wise correlations between structural network *metrics *and averaged functional *metrics *extracted from fast time scale networks. The structure-function similarity in local clustering and the discrepancy in global closeness at the asymptotic state (Figures 3, 4) are reflected in the corresponding correlations between node-wise clustering (Figure 7B) and closeness (Figure 7C). Correlations between other network metrics (Additional file 2) are intermediate to these two extremes. No such correlations are present in the initial networks, while the correlations in evolving networks are qualitatively different – as illustrated by the transient early anticorrelation between clustering (Figure 7B).

There also exists an alternative approach to extracting correlations from structural and functional networks. This involves exchanging the sequence of our initial analysis by firstly calculating correlations between the structural and fast time scale functional networks, and subsequently temporally averaging these correlations. This second approach emphasizes the instantaneous expression of structure-function correlations. Figure 7A–C shows that correlations obtained in this way are significantly weaker. Figure 7D shows the gradual increase in correlation strengths that accompanies the transition from a fast, to a slow time scale analysis, at the asymptotic stage of rewiring.

### The dynamics of central and peripheral nodes

**Figure 8:**
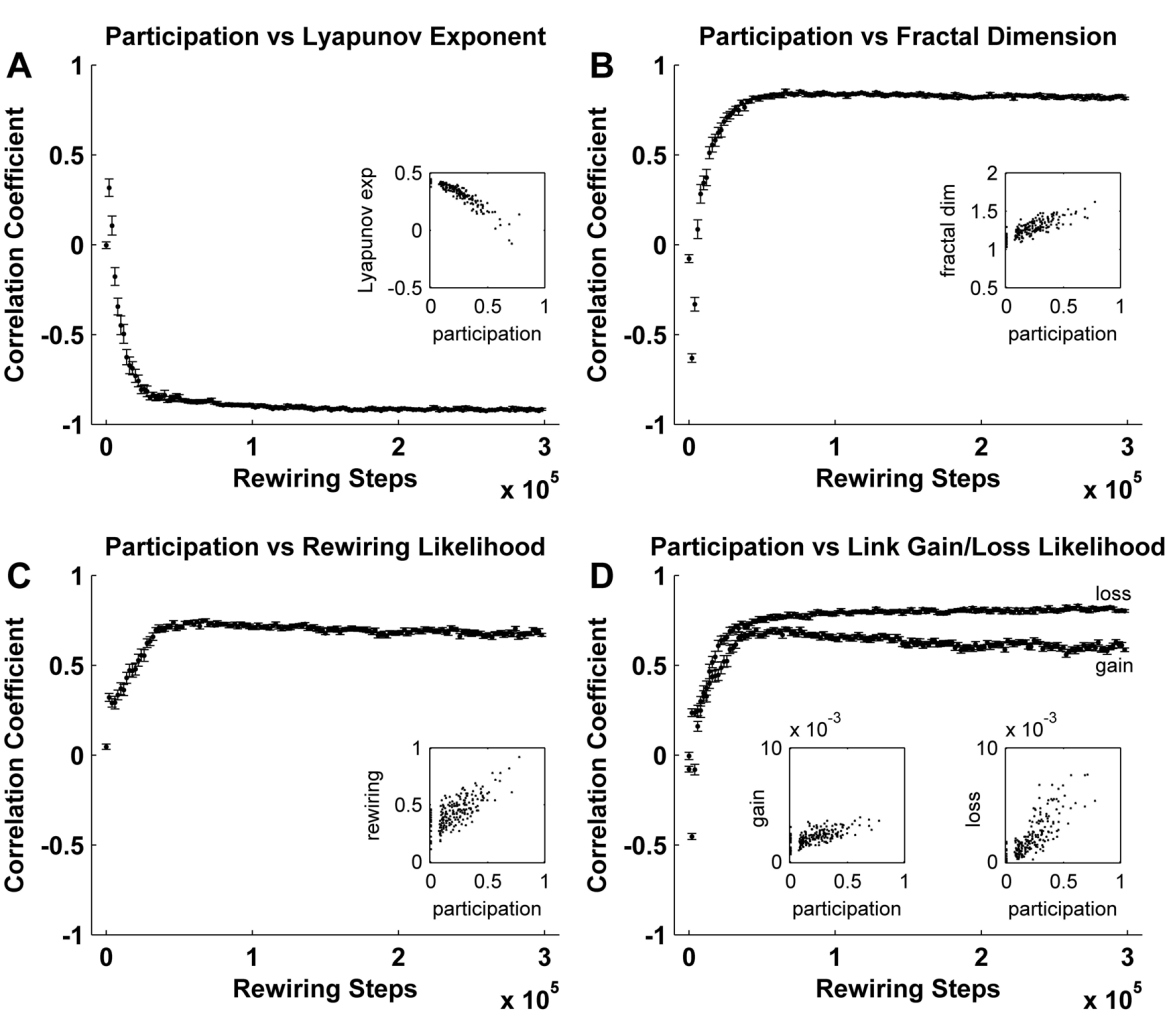
**Correlation between centrality, dynamics and rewiring**. Temporal evolution of the correlation between participation and Lyapunov exponent (A), fractal dimension (B), rewiring rate (C), and the likelihood of losing or gaining a link to a rewirable node (D). Scatter plots illustrate typical correlations at the asymptotic state. Participation is a measure of centrality, sensitive for nodes with connections distributed over multiple modules. Note that participation is unreliable at the early stages of evolution, given the weakly modular nature of structural networks. Error bars represent the standard error of the mean, as estimated over 20 simulations.

Central and peripheral nodes manifest distinctly different dynamics (Figure 8A,B). We define nodes to be "central" or "peripheral" according to their connection patterns. Specifically, nodes are said to be peripheral when they mostly connect with nodes in their own module (low participation) and central when they mostly connect with nodes in other modules (high participation). Peripheral nodes receive homogeneous inputs, and consequently exhibit high synchrony and low-dimensional chaotic dynamics. On the other hand, central hubs connect with nodes in multiple modules, receive discordant inputs, and consequently exhibit unsynchronized, high-dimensional stochastic dynamics. Noisy hub dynamics correspond to high rewiring likelihoods (Figure 8C), with a high chance of losing or gaining a connection when rewiring occurs (Figure 8D). In addition, within hub populations, nodal degree positively correlates with link loss, and negatively correlates with link gain (Additional file 3). These findings hence suggest that at the asymptotic state, rewiring largely occurs between hubs, in a cyclical-like pattern.

**Figure 9:**
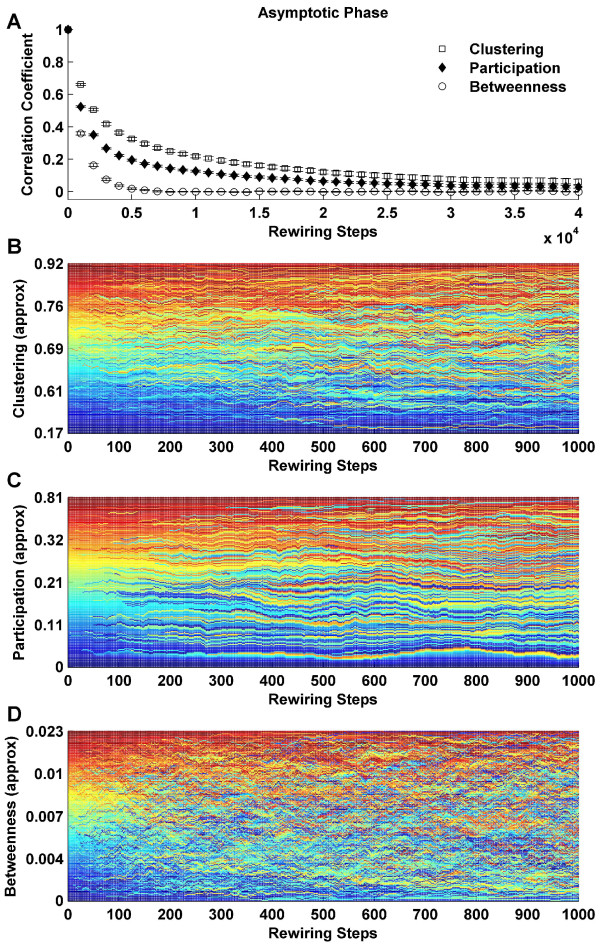
**Fluctuation of structural centrality metrics at the asymptotic phase**. (A) Node-wise autocorrelation of clustering, participation and betweenness at the asymptotic phase as a function of progressive rewiring. Error bars represent the standard error of the mean, as estimated over 20 simulations. (B-D) An illustration of the rapid fluctuations in clustering (B), participation (C) and betweenness (D). Note the shorter time scale, compared to (A). Nodes were rank-ordered by centrality (from lowest to highest) at each rewiring step. Color corresponds to the rank-ordering position at the first sampled rewiring step.

Continuous network plasticity gradually "mixes" individual structural metrics across the network, even though the network-wide metric averages remain invariant. Figure 9A shows the gradual decorrelation of node-wise structural metrics as a function of rewiring steps. Centrality indices continually fluctuate, and decorrelate more rapidly than clustering. Figure 9B–D shows exemplars of this mixing of node-wise metrics at the asymptotic stage of evolution, when the topology is globally invariant. In these panels, nodes were rank-ordered at the first sampled time step and then assigned a rank-specific color. At subsequent steps, nodes were reranked and therefore reordered, but the color-coding remained fixed. The mixing of colors hence represents fluctuations in rank positions. For example, red strips in the midst of deep blue in Figure 9C, correspond to nodes which have significantly dropped their centrality rank over the sampled interval.

**Figure 10:**
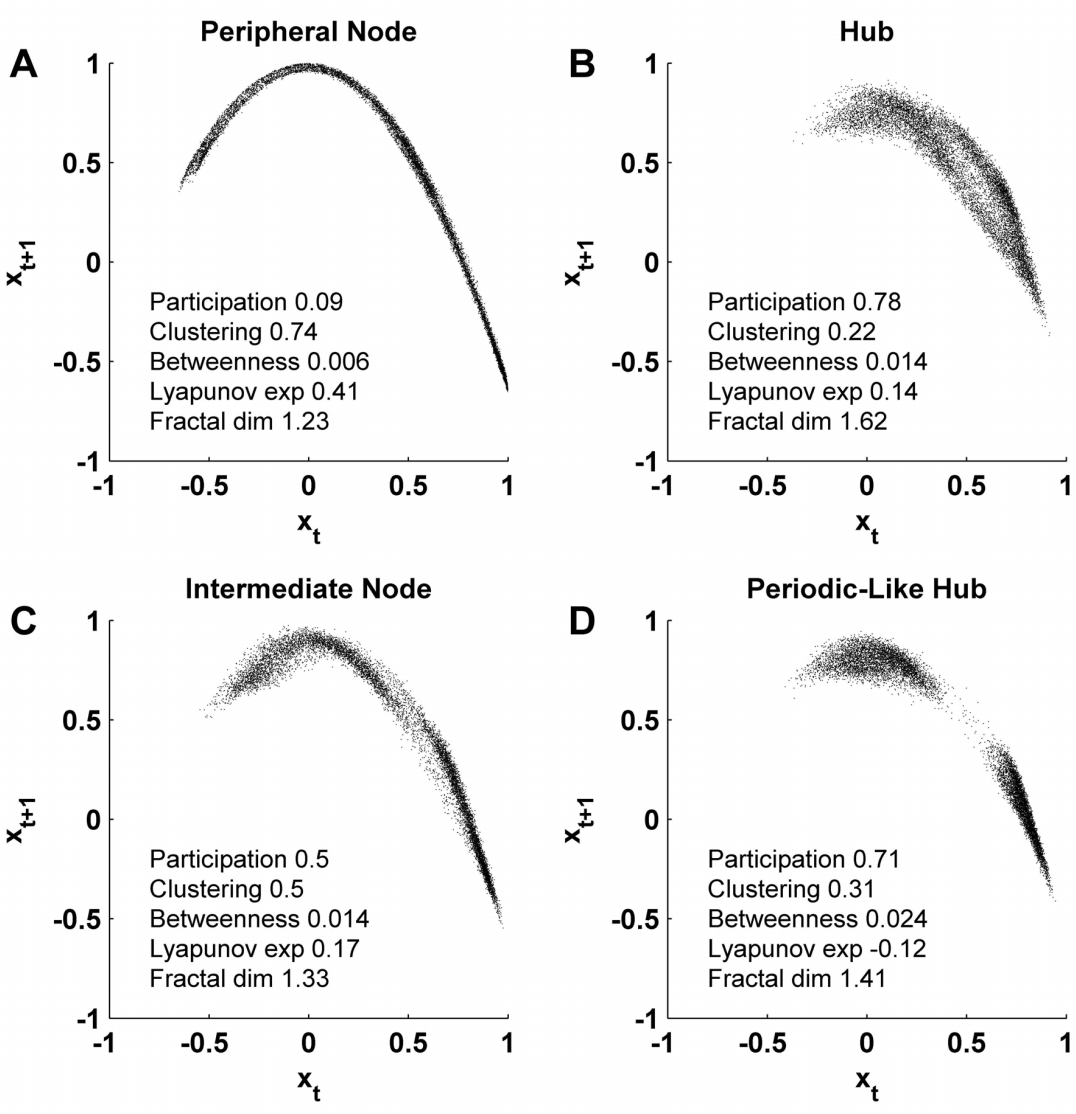
**Maps of four representative nodes**. (A) Low-dimensional chaotic dynamics of a peripheral node, (B) Stochastic high-dimensional cloud of a highly participating hub, (C) An intermediate node, whose dynamics resemble the Poincaré first return map of the neural mass model in Figure 2B, (D) Contracting dynamics of a periodic-like node, perturbed by unsynchronized inputs.

Figures 10A and 10B show representative maps for peripheral and central nodes, illustrating the respective thin and cloud-like patterns. Figure 10C shows the dynamics of an intermediate node, whose map resembles the Poincaré return map of the neural mass model in Figure 2B. Figure 10D shows the dynamics of a node with (noise perturbed) periodic activity which exhibits contracting dynamics, and therefore a negative Lyapunov exponent.

## Discussion

The elusive nature and role of structural and functional brain connectivity [42] is a frontier topic in systems neuroscience [43]. While empirical studies, aided by computational techniques, provide fascinating insights into development and maturation of brain networks [44], modeling studies allow the causal mechanisms behind such development and maturation to be parametrically explored. We explored a simple, biophysically motivated model, to probe the underlying mechanisms of large scale neuroanatomical self-organization. We observed that random structural connectivity is reshaped by ordered functional connectivity towards a modular topology. However, in spite of the order in functional networks, structural topology remains robustly interconnected, and therefore small-world, due to the noisy dynamics of central hub nodes.

Our construction of functional networks is based on the calculation of Euclidean distance between one-dimensional unit states (see Methods), and will necessarily generate ordered fast time scale functional connectivity, no matter how chaotic the dynamics. More importantly, however, functional networks constructed on a slower time scale likewise remain ordered (Figures 4, 5), suggesting that the topology of synchronous connections repeatedly recurs. These recurring ordered functional topologies reflect clustered synchronization of the coupled chaotic dynamics [35]. By varying the parameters we note that structural evolution to a clustered topology occurs most prominently under chaotic dynamics and moderate coupling – hence, there must be sufficient homogeneity (due to moderate coupling) in order to enable the formation of synchronous functional clusters, but also sufficient variability (due to chaotic dynamics) to enable the emergence of multiple such clusters.

On a random structural network, synchrony is likely to be stronger between nodes with chance higher connectivity. It is probable that early in neuroanatomical development, higher connectivity strongly correlates with spatial proximity. We find that such connectivity is subsequently reinforced by activity-dependent rewiring; a process which leads to the emergence of clustered structural modules. Therefore, in our simulations, functional networks emergent on random structural networks, anticipate the asymptotic modular connectivity. Our model illustrates a potential mechanism by which brain-like structural connectivity may emerge in an unsupervised way, without a global search for optimal connectivity. It is known that a global search (testing all possible synapses) is a hard combinatorial optimization problem in a sparse network [11], and is hence unlikely to occur *in vivo*.

We find that slow time scale functional connectivity strongly reflects the underlying structural connectivity, in agreement with recent reports [23-25]. Functional networks fluctuate at faster time scales, but gradually become more stable and constrained by structure at slower time scales. The fluctuation of fast time scale functional connectivity may be enabled by the presence of structural hubs. These nodes interconnect multiple modules, and consequently preserve a small-world structural topology in the face of ordered functional connectivity. Structural hubs may therefore enable the delicate interplay between the segregation and integration of functionally specialized processing, which is thought to represent the hallmark of functional brain complexity [8,30]. The rapidly expanding network-based analyses of structural connectivity in empirical data have already identified candidate hub locations and their putative roles [5,45].

The present theoretical approach may also be used to interpret functional connectivity findings from empirical studies, by validating structural connectivity patterns against DTI data, and validating functional connectivity patterns against EEG or MEG data (on fast time scales) and fMRI data (on slower timescales). For example, a detailed classification of hubs in mammalian neuroanatomical networks has recently been performed [45], but the nature of the dynamics of these hubs has not been studied. Whilst we do not explicitly explore this approach through forward modeling (such as applying a neurovascular model to neuronal states [25]), such an implementation would be relatively straightforward.

The role of noise in neural systems is currently a subject of considerable interest [46]. We heuristically defined noise to correspond to high-dimensional dynamics arising from uncorrelated, but ultimately deterministic inputs (Figure 10B) – in this way our definition is conceptually different from network models with an explicit stochastic component [47]. The dynamics on a random network show very little noise with near global synchronization. At the asymptotic state, most nodes exhibit low-dimensional chaotic dynamics, because they are embedded in densely interconnected modules with homogeneous intra-modular dynamics. A smaller number of hub-like nodes exhibit high-dimensional noisy dynamics. Noise in this system can hence be seen to have attained a strong topological structure, being generated within the network and then "directed towards" the few central hub nodes. This high-dimensional activity allows such hubs to explore the dynamical neighborhoods of other nodes, participate as both connection donors and recipients, and ensure the system remains structurally connected. Noise in this system is highly organized and is an emergent feature of the global dynamics.

We also explored the influence of slower time scale dynamics on activity-dependent rewiring, by incorporating a memory function into the rewiring rule. Such a function may represent a gradual consolidation of memories in cortical tissue. However, the use of a memory function which linearly decays with time is putatively problematic, given that the resulting slow time scale networks neglect any itinerant dynamics and consequently fail to capture the richness of instantaneous functional states (Additional file 4). Future studies would benefit from a memory function that could capture the multiscale temporal character of functional connectivity, such as a sequence of coupled exponential functions [48]. Such an approach may also reveal small-world functional networks, in accordance with a multitude of recent empirical reports [9,10,41,49,50].

A clear neurobiological limitation of the present study is the use of the simple unimodal map. We have provided a cursory justification for this by comparing the Poincaré first return map of a detailed neural mass model with the unimodal topology of the logistic map (Figure 2). Furthermore, the neural mass model and logistic map both share the same underlying dynamical mechanisms, namely homoclinic chaos around a single fixed point. Kwok et al. [22] have also observed evolution to a small-world using the same adaptive rewiring rule but with a Hindmarsh-Rose spiking neuron model. However, in order to provide a more detailed analysis of the structure-function-dynamics tripartite, we returned to the logistic map to enable a computationally parsimonious derivation of structural and functional measures, and explicit calculation of invariant measures of dynamical behavior such as the Lyapunov exponent. Such an approach lays the groundwork for more exact descriptions of phenomena in complex dynamical models, with consequent functional interpretations of specific biophysical dynamics such as bursting.

## Conclusion

We explicitly conceptualized the interdependent relationship between structural and functional brain connectivity, and explored the mechanisms by which this relationship may lead to the emergence of cortical-like structural networks. Our study theoretically reinforces the central role for neuronal dynamics in the emergence of complex brain connectivity. We show that functional connectivity becomes gradually more constrained by the underlying structural connectivity, as functional networks are extracted at increasingly slower time scales. The fluctuations of functional networks at faster time scales may arise from the noisy dynamics of central structural nodes.

## Methods

### Structural and dynamical components of the model

The model consists of an ensemble of quadratic logistic maps, coupled via a directed binary connectivity matrix. Following established neuroscientific notation, we refer to the coupling matrix as structural connectivity. Correspondingly, we refer to the correlations between dynamical states arising on structural connectivity, as patterns of functional connectivity [51].

Formally, we represent structural connectivity with a directed binary graph *G *= ⟨*N*, *L*⟩, consisting of *N*, the set of *n *nodes, and *L*, the set of *l *directed links (edges, connections) between pairs of nodes. In our simulations, *n *= 200 and *l *= 4000, corresponding to 10% connectedness. *G *may be also defined by a corresponding connectivity matrix *A*, in which a node *i *is said to neighbor node *j *(*i*, *j *∈ *N*), when there exists a directed connection from *i *to *j*, as represented by *a*_*ij *_= 1; the lack of such a connection is denoted by *a*_*ij *_= 0 (with self-connections not allowed by definition). Let *N*_*i *_represent the set of neighbors (neighborhood) of node *i *and let *n*_*i *_be the number of neighbors (degree) of *i*; correspondingly let the complement  represent the set of all non-neighbors of *i*.

The set of nodes *N *has a corresponding dynamical ensemble **X**; hence each node *i *has a corresponding dynamical unit *x*_*i*_. The dynamics of the unit state at discrete time *t *are governed by a commonly used quadratic logistic map



where the control parameter *μ *governs the nature of the dynamics (0 ≤ *μ *≤ 2). The neural ensemble is constructed through coupling these maps, as



where coupling is facilitated through in-connections and *ε *represents coupling strength (0 ≤ *ε *≤ 1). Unit states were initially assigned random values (-1 ≤ *x*_*i *_(0) ≤ 1 for all *x*_*i *_∈ **X**).

The coupling parameter *ε *may be thought to represent the neuromodulatory influence of diffusively projecting brainstem monoamine neurons on the synaptic efficacy of corticocortical fibers [52]. The parameter *n*_*i *_effectively rescales the coupling input, and may be thought to represent the mechanisms of homeostatic neuroplasticity [53]. Such mechanisms maintain relatively constant firing rates in the face of variable synaptic input, and hence play an important stabilizing role in networks with a nonhomogeneous connectivity distribution. In our model, unit states diverged to infinity in a significant number of simulations conducted without rescaling.

The control parameter *μ *varies the degree of nonlinearity (the curvature of the quadratic hump) in each node. Mathematically, *μ *acts as a simple one-dimensional bifurcation parameter for intra-node dynamics. Nonlinear effects in neural models, such as the neural mass model of Figure 1, arise from the highly nonlinear nature of voltage-gated membrane channels, and hence from the excitable neural membrane. At the level of a single channel, this effect is a step-function. At the level of a neuron, or a population of neurons, the step-function is smoothed by the threshold variance of individual channels in the population. The smaller the variance, the stronger the effective nonlinearity, as characterized by a higher value of *μ*. Following Gong and van Leeuwen [20], we initially set *μ *= 1.7 and *ε *= 0.5, hence enabling chaotic dynamics and moderate coupling. Subsequently, we investigated the robustness of our results across a range of parameter values.

### Activity-dependent rewiring rule

We used a rewiring rule which periodically modified the structural connectivity matrix towards emergent patterns of functional connectivity. For each structural network, the dynamics were iterated for 1000 iterations. Following this, a node was randomly chosen and its connections were rewired such that it gained a link to a node with which it was most synchronous, and lost a link to a neighbor with which it was least synchronous. If the most synchronous node was already a neighbor, a different node was chosen until one connection was successfully rewired. This rule exploits the fact that all nodes have identical parameter values so that the Euclidean distance |*x*_*i *_- *x*_*j*_| accurately captures pair-wise synchronization.

Formally, a node *i *∈ *N *was deemed rewirable when there existed a non-neighbor *k *∈  that minimized |*x*_*i *_- *x*_*N*_|; that is, when *i *was not connected to a node with which it was most synchronous. For this *i*, a neighbor *j *∈ *N*_*i *_was chosen, such that *j *maximized ; that is, such that *j *was the least synchronized neighbor of *i*. The connections were then rewired as *a*_*ik *_= 1 and *a*_*ij *_= 0. Rewiring alternated between in and out neighbors at consecutive steps.

### Extraction of functional networks

For each structural topology, "fast time scale functional networks" were extracted through computing inter-unit synchrony – measured as the Euclidean distance between instantaneous dynamical unit states. A strongest synchrony threshold was applied to convert the resulting synchrony matrices into binary networks, of the same connection density as the structural networks. For a given structural network, we extracted an ensemble of fast time scale functional networks and characterized their properties using network analysis methods. We then averaged the resulting metrics over time to obtain characteristic functional network metrics expressed on a given structural state. Hence we first extracted network metrics from fast time scale networks, and subsequently averaged these metrics. This contrasts with an alternative approach, whereby an ensemble of fast time scale networks is first averaged, and network metrics are subsequently extracted from the resulting "slow time scale networks". The two approaches are not commutative. We focused on the first approach, which emphasizes the average expression of spatiotemporal dynamics in functional connectivity, but also permits incorporating the effects of transient synchrony. Such itinerant effects are averaged out in the slow time scale functional networks.

Formally, fast time scale functional networks were constructed from **X **as *n *× *n *symmetric synchronization matrices, where each entry (*i*, *j*) corresponded to |*x*_*i *_- *x*_*j*_|. For a given structural network, one functional network was extracted at every tenth iteration of the dynamics, hence enabling an ensemble of 100 fast time scale functional networks for 1000 iterations. Each network was individually analyzed, and the obtained network metrics were averaged to represent the characteristic functional topology. In the initial random networks, all unit states rapidly synchronized, and the dynamics were hence iterated only while there existed a meaningful difference between states (typically for 400–500 iterations, hence enabling the extraction of 40–50 functional networks). Slow time scale functional networks were extracted by averaging 100 consecutive fast time scale functional networks.

### Network analysis methods

We analyzed structural and functional connectivity properties using metrics of local and global network topology, as well as of individual node centrality. All computations were performed in Matlab (The MathWorks, Inc.), using double precision arithmetic. Our network analytic software is available to download from .

The clustering coefficient for an individual node, represents the likelihood that any two neighbors of that node will themselves be neighbors [1]. For an undirected network the average clustering coefficient is given by



We computed the directed clustering coefficient using the method of Fagiolo [54].

Closeness represents the average distance from one node, to all other nodes in the network [55]. We calculated closeness as a harmonic mean of the shortest path length [56]. This definition allows to calculate distances on disconnected networks. The average closeness for the network is given by



where *d*_*ij *_is the shortest path length between nodes *i *and *j*.

Small-world networks are defined as networks that are significantly more clustered than surrogate random networks (*C*/*C*_*random *_>> 1), but have approximately the same closeness as random networks (*E*/*E*_*random *_≈ 1). Surrogate random networks were generated using the degree distribution preserving algorithm of Maslov and Sneppen [57].

Modularity describes the presence of groups of nodes (modules) which have dense intra-group connectivity, but only sparse inter-group connectivity. We subdivided the network into a set of modules *M *using the spectral optimization algorithm of Newman [7], generalized to directed networks [58]. The optimized modularity *Q *hence reflects the strength of modular structure, by contrasting the density of intra-module to inter-module connections. The modularity is defined as



where *e*_*uv *_represents the proportion of all links in the network that connect nodes in module *u *to nodes in module *v*.

Node centrality was assessed with the participation coefficient [59]. Participation measures the heterogeneity of nodal connections across modules – highly participating hubs are defined as nodes which connect to a large number of modules. Participation is closely related to other measures of centrality such as the flow coefficient [25] and local betweenness centrality [60]. In our simulations participation was moderately well correlated with betweenness centrality, strongly anticorrelated with the clustering coefficient, and more sensitive than betweenness to detecting the number of inter-modular interconnections (Additional file 5). The participation coefficient for an individual node *p*_*i *_is defined as



where *η*_*iu *_is the number of links between node *i *and nodes in module *u*. We calculated the participation coefficient for in-neighbors, in symmetry with in-neighbor coupling of the logistic maps.

Nodal rewirability was estimated for each structural network by comparing the network with a corresponding ensemble of functional networks, emergent on that structural connectivity.

### Nonlinear dynamical analysis

We characterized the temporal dynamics of individual units by computing their Lyapunov exponents and the fractal dimensions of their corresponding attractors. Taken together, these metrics indicate whether the dynamics are chaotic and low-dimensional (positive Lyapunov exponent and low fractal dimension), or alternatively, due to discordant inputs, are better characterized as high-dimensional and stochastic. Note that, given the deterministic nature of the logistic map, we use the term stochastic heuristically, to invoke the putative impact of multiple uncorrelated chaotic inputs via the coupling term.

Formally, the Lyapunov exponent for an individual unit *x*_*i*_, denoted as *λ*_*i*_, quantitatively determines the average stability of the orbit of the attractor of *x*_*i*_. The Lyapunov exponent was approximated as



where *μ *is the control parameter of the logistic map and *T *= 1000 denotes the number of iterations of the dynamics at each rewiring step.

The fractal (correlation) dimension for an individual unit *x*_*i*_, denoted as *D*_*i*_, estimates the dimension of the attractor of *x*_*i *_[61]. The fractal dimension is given by



where *C*(*r*) is the average number of points in the attractor within a ball of radius *r*. *D*_*i *_was approximated by generating 1000 points of the orbit of *x*_*i *_and computing *C*(*r*) for 50 randomly chosen points, with 0.01 ≤ *r *≤ 0.3. Plots of log(*C*(*r*)) versus log(*r*) were visually inspected to ensure the presence of a robust linear relationship.

## Authors' contributions

MR, OS, CVL, MB designed research. MR, MB performed research. MR, MB analyzed the data. MR, OS, CVL, MB contributed reagents/materials/analysis tools. MR, MB wrote the paper. All authors read and approved the final manuscript.

## Supplementary Material

Additional file 1 **Evolution of degree in structural and functional networks**. Minimum and maximum degree, along with the mean and standard deviations (dotted lines) for structural (black) and functional (blue) networks. Error bars represent the standard error of the mean, as estimated over 20 simulations. Click here for file

Additional file 2 **Correlation between structural and functional network metrics**. Temporal evolution of the correlation coefficient between structural and functional participation (A), betweenness (B) and degree (C) with illustrative scatter plots (insets) at specified time instants. Functional network metrics are derived by averaging the metrics of fast time scale networks. An alternative approach, emphasizing the instantaneous expression of functional connectivity (see text) results in significantly weaker correlations (solid lines). Error bars represent the standard error of the mean, as estimated over 20 simulations. Click here for file

Additional file 3 **Correlation between degree and the likelihood of link gain or loss**. Temporal evolution of the correlation coefficient between degree and link gain/loss likelihood for all nodes (A), and for central nodes only (B), defined as those nodes with participation of greater than 0.4. Error bars represent the standard error of the mean, as estimated over 20 simulations. Click here for file

Additional file 4 **Relationship between fast time scale and slow time scale functional connectivity**. (A) Five consecutive iterations of spatiotemporal dynamics are shown in the top row, with the corresponding functional networks in the bottom row, ordered by the corresponding structural modular arrangement. Note the complex interplay of intra and inter-modular synchrony, reflecting a mix of segregative and integrative dynamics. (B) Dynamics and functional network obtained by calculating the correlation coefficient for the five iterations in A. The inter-modular synchrony is largely averaged at this slower time scale. Click here for file

Additional file 5 **Correlation between participation and other structural network metrics**. Temporal evolution of the correlation coefficient between participation, and betweenness centrality (A), clustering (B), degree (C), and the number of modules interconnected by a node (D). Scatter plots illustrate typical correlations at the asymptotic state. Error bars represent the standard error of the mean, as estimated over 20 simulations. Click here for file
