# Supplementary figures and images for: Symbiotic relationship between brain structure and dynamics
Source: BMC Neurosci. 2009 Jun 2;10:55. doi: 10.1186/1471-2202-10-55 (PMC2700812; doi:10.1186/1471-2202-10-55)

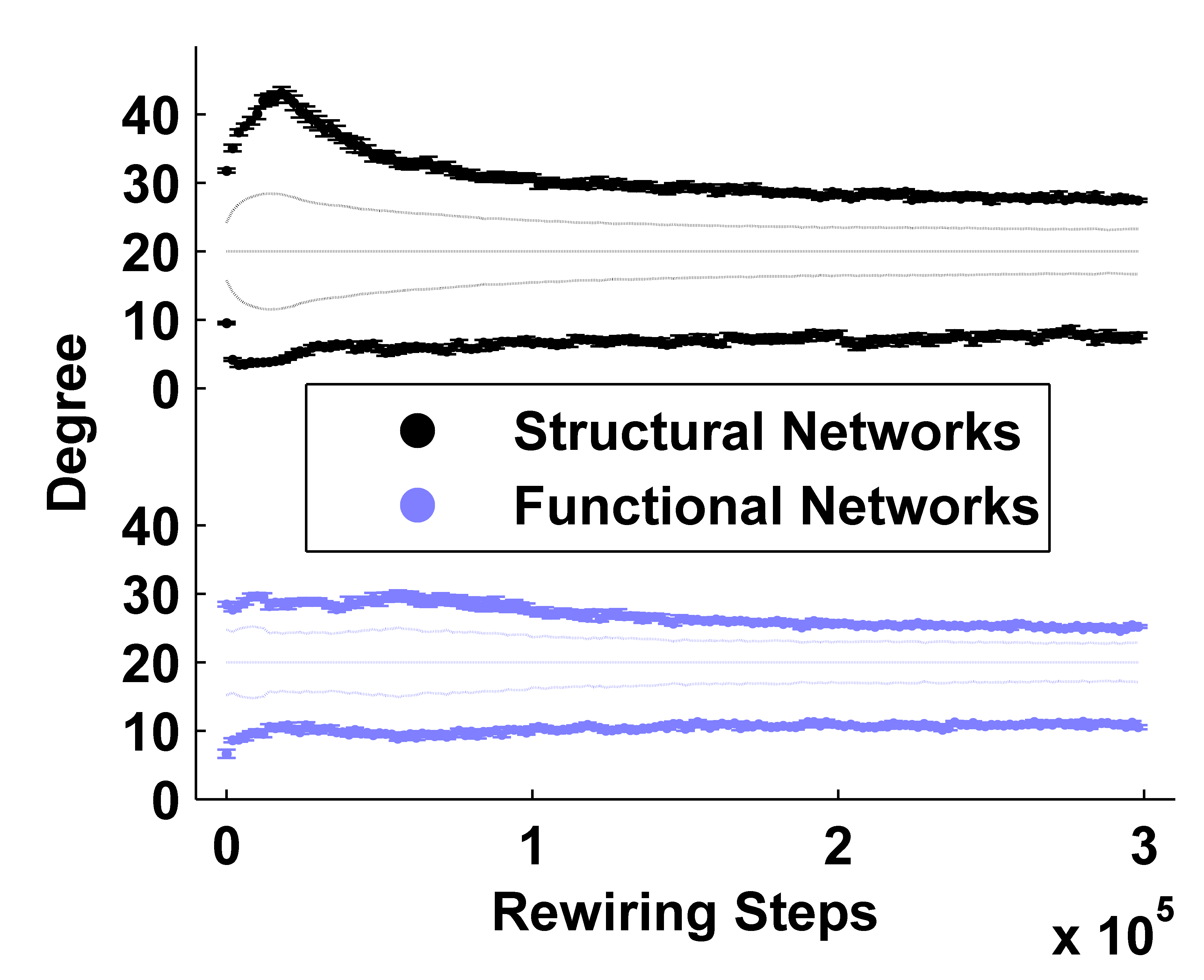

Supplement: Additional file 1 — Evolution of degree in structural and functional networks. Minimum and maximum degree, along with the mean and standard deviations (dotted lines) for structural (black) and functional (blue) networks. Error bars represent the standard error of the mean, as estimated over 20 simulations. [file 1471-2202-10-55-S1.png]

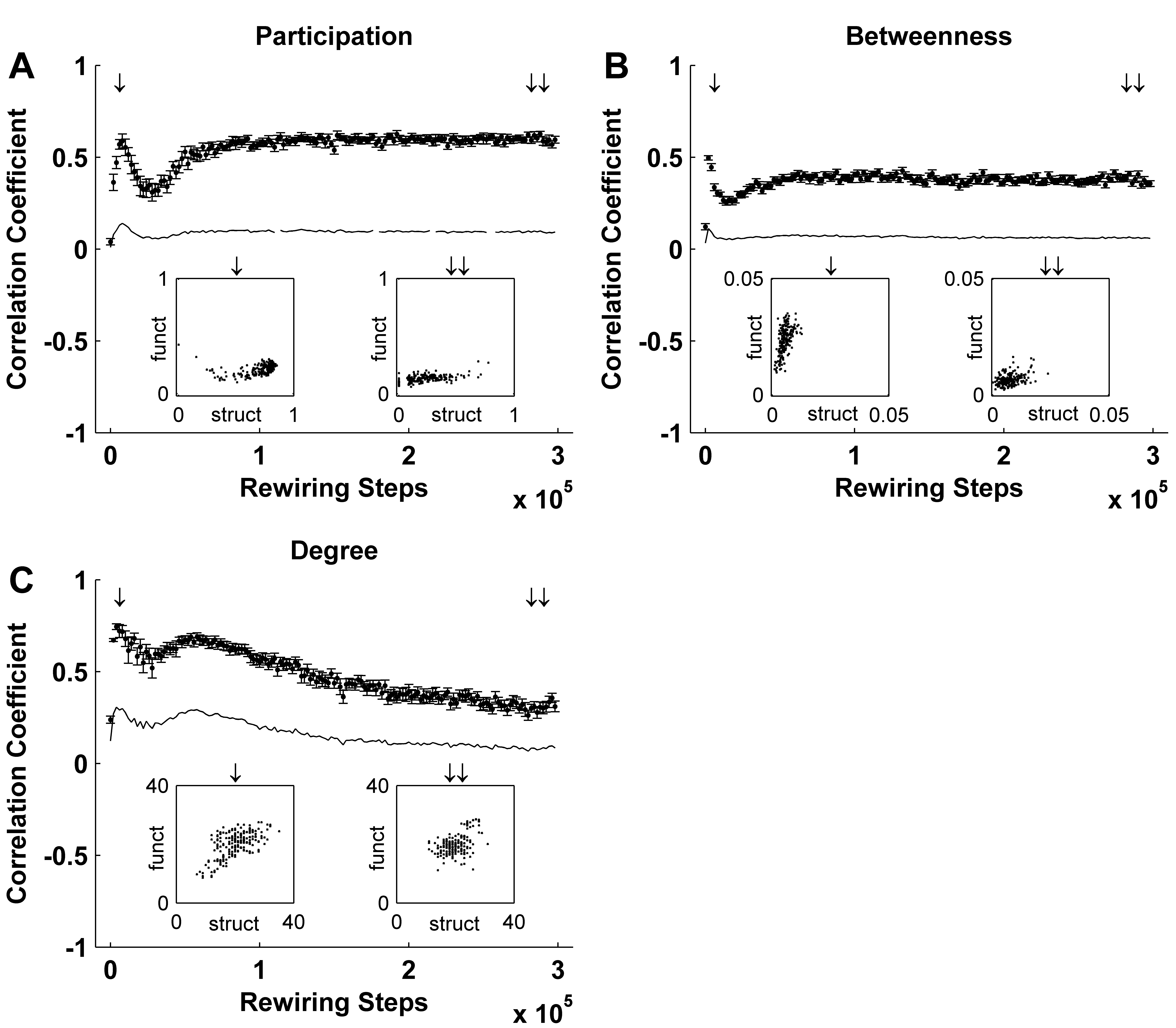

Supplement: Additional file 2 — Correlation between structural and functional network metrics. Temporal evolution of the correlation coefficient between structural and functional participation (A), betweenness (B) and degree (C) with illustrative scatter plots (insets) at specified time instants. Functional network metrics are derived by averaging the metrics of fast time scale networks. An alternative approach, emphasizing the instantaneous expression of functional connectivity (see text) results in significantly weaker correlations (solid lines). Error bars represent the standard error of the mean, as estimated over 20 simulations. [file 1471-2202-10-55-S2.png]

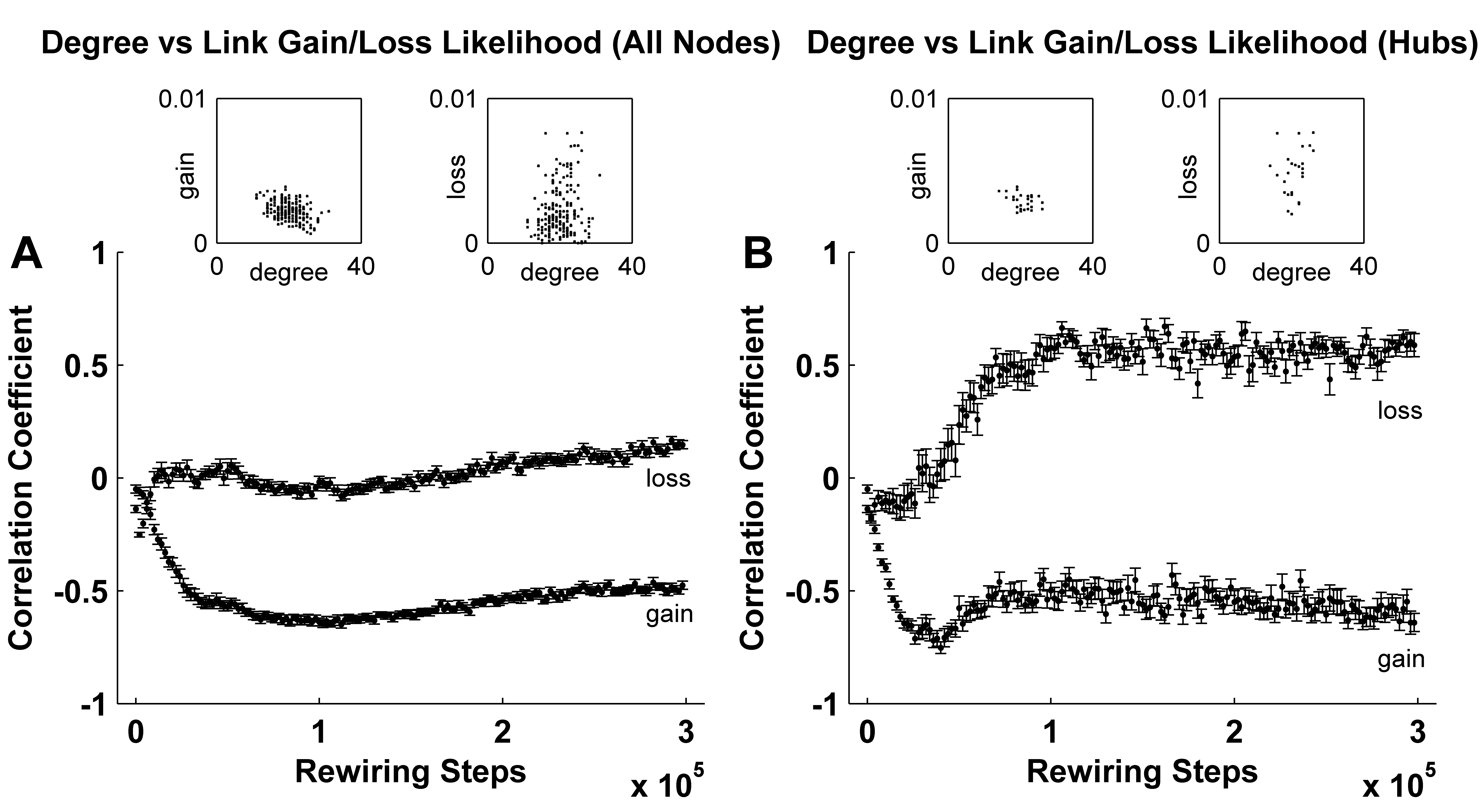

Supplement: Additional file 3 — Correlation between degree and the likelihood of link gain or loss. Temporal evolution of the correlation coefficient between degree and link gain/loss likelihood for all nodes (A), and for central nodes only (B), defined as those nodes with participation of greater than 0.4. Error bars represent the standard error of the mean, as estimated over 20 simulations. [file 1471-2202-10-55-S3.png]

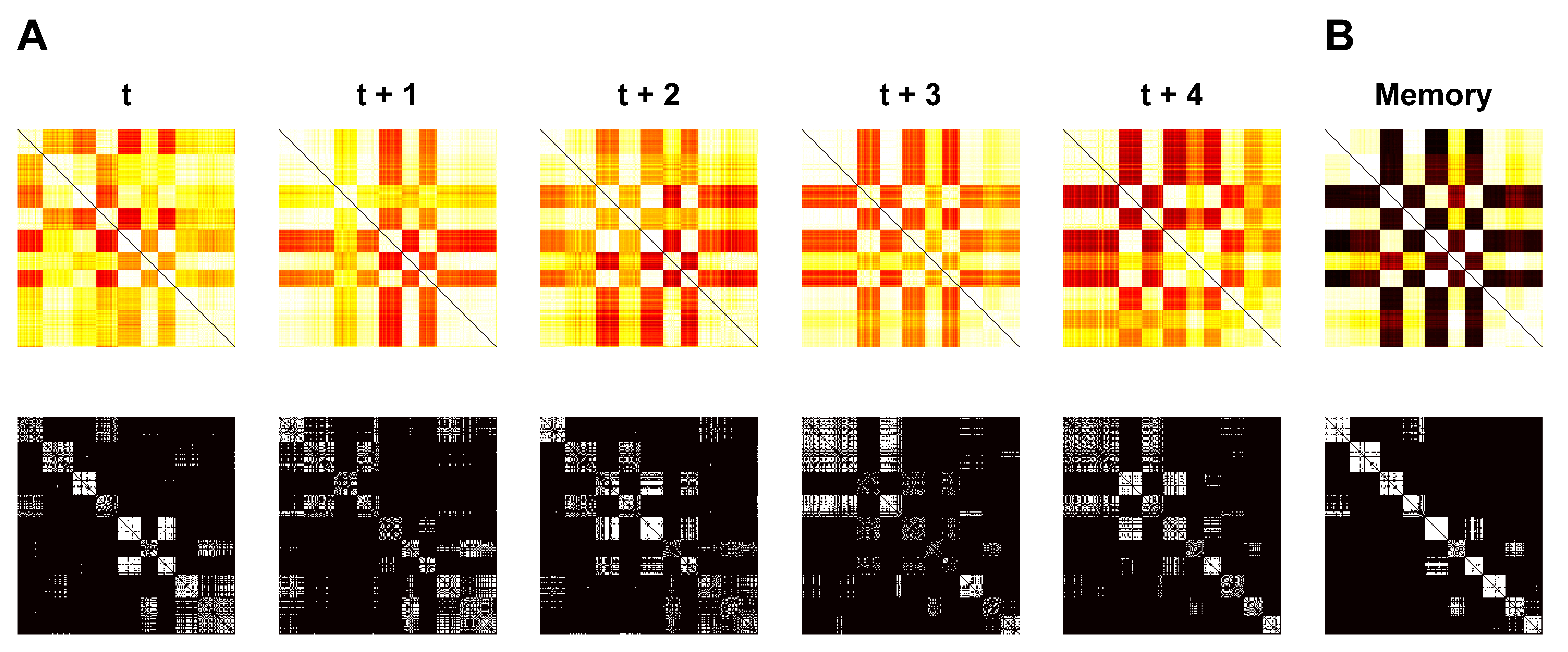

Supplement: Additional file 4 — Relationship between fast time scale and slow time scale functional connectivity. (A) Five consecutive iterations of spatiotemporal dynamics are shown in the top row, with the corresponding functional networks in the bottom row, ordered by the corresponding structural modular arrangement. Note the complex interplay of intra and inter-modular synchrony, reflecting a mix of segregative and integrative dynamics. (B) Dynamics and functional network obtained by calculating the correlation coefficient for the five iterations in A. The inter-modular synchrony is largely averaged at this slower time scale. [file 1471-2202-10-55-S4.png]

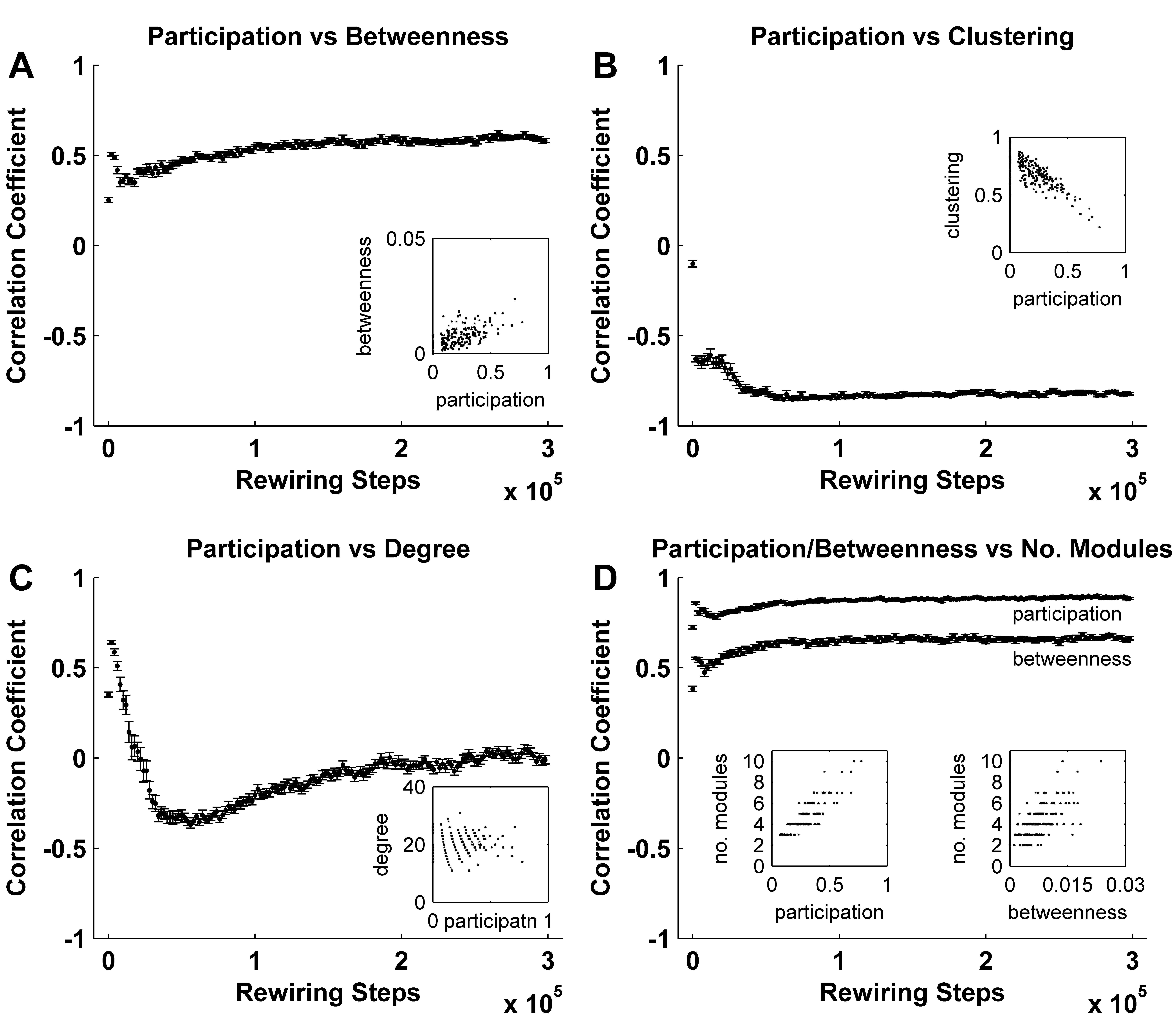

Supplement: Additional file 5 — Correlation between participation and other structural network metrics. Temporal evolution of the correlation coefficient between participation, and betweenness centrality (A), clustering (B), degree (C), and the number of modules interconnected by a node (D). Scatter plots illustrate typical correlations at the asymptotic state. Error bars represent the standard error of the mean, as estimated over 20 simulations. [file 1471-2202-10-55-S5.png]
